# 1034. FebriDx use in Immunocompromised Patients in a Real-World Hospital Setting during the second (COVID-19) wave in Italy

**DOI:** 10.1093/ofid/ofab466.1228

**Published:** 2021-12-04

**Authors:** Filippo Lagi, Alessandro Bartoloni, Catalina Suarez-Cuervo

**Affiliations:** 1 Department of experimental and clinical medicine, Florence, Toscana, Italy; 2 Department of Experimental and Clinical Medicine, University of Florence, Florence, Italy, Florence, Toscana, Italy; 3 Lumos Diagnostics, Lakeland, Florida

## Abstract

**Background:**

The diagnosis of acute respiratory infection (ARI) in patients with immunosuppression secondary to disease or medications is often unclear. Symptoms may be absent or blunted, and acute phase reactants, like procalcitonin (PCT) and C-reactive protein (CRP) may not elevate. For these patients, minor signs or symptoms could lead to hospitalization and antibiotic prescriptions to prevent complications or death. FebriDx® is a rapid, qualitative immunoassay test designed to distinguish between viral or bacterial respiratory infection through simultaneous detection of both CRP and Myxovirus resistance protein A (MxA) from a fingerstick blood sample.

**Methods:**

FebriDx was evaluated as part of a real-world prospective, observational study in hospitalized patients with symptoms of ARI and suspected COVID-19 in a single tertiary care center in Italy (August, 2020 - January, 2021). A sub analysis of patients with expected reduced host immune responses secondary to immunosuppression by disease or medication was performed. (Classified by treating clinician; patient on high dose steroids/ immunosuppressive therapy, or underlying condition like cancer or autoimmune disease). Sensitivity, specificity, positive predictive value (PPV), negative predictive value (NPV), and likelihood ratios were calculated for FebriDx with respect to the final diagnosis.

**Results:**

We included 28 patients from 200 in the study, 16 patients had a final diagnosis of bacterial infection and 12 had viral infection. FebriDx showed a sensitivity of 91.7% to accurately diagnose viral infection and 93.8% for bacterial infection (see tables). Serum CRP was not available for 4 of the patients included (14%) and elevated in the remaining patients. PCT was not available for one patient with viral infection and was elevated in 50.0%.

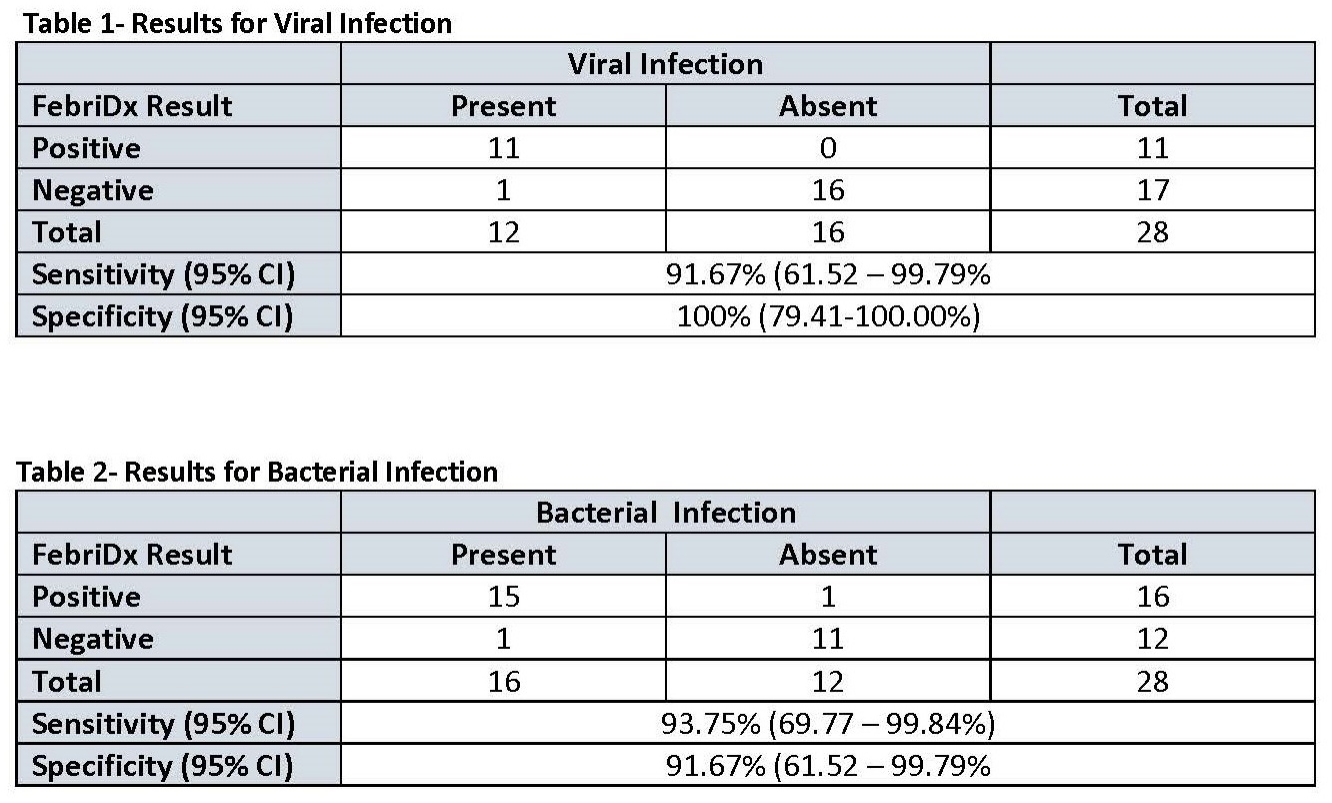

FebriDx Performance when compared to Clinical Diagnosis

**Conclusion:**

FebriDx demonstrated a higher accuracy for differentiating bacterial vs. viral infection in an immunocompromised cohort than single biomarkers CRP and PCT. FebriDx demonstrated a high diagnostic accuracy to differentiate viral from bacterial infection in patients with chronic immunosuppressive conditions in a real-world setting and had better performance than standalone CRP and PCT to distinguish viral and bacterial ARI in immunocompromised patients.

**Disclosures:**

**Catalina Suarez-Cuervo, MD**, **Lumos Diagnostics** (Employee)

